# Supplementary material for: End Sequence Analysis Toolkit (ESAT) expands the extractable information from single-cell RNA-seq data
Source: Genome Res. 2016 Oct;26(10):1397–410. doi: 10.1101/gr.207902.116 (PMC5052061; doi:10.1101/gr.207902.116)
Supplement: Supplemental Material [file supp_gr.207902.116_Supp_Materials.tar.gz › ESAT-esat_v0.1/doc/html/classbroad_1_1pda_1_1datastructures_1_1_alignments.html]

ESAT: broad.pda.datastructures.Alignments Class Reference


ESAT


- Main Page
- Related Pages
- Packages
- Classes
- Files

- Class List
- Class Index
- Class Hierarchy
- Class Members

All Classes Namespaces Files Functions Variables Enumerator Pages

- broad
- pda
- datastructures
- Alignments

Public Member Functions |
Static Public Member Functions |
List of all members

broad.pda.datastructures.Alignments Class Reference

Inheritance diagram for broad.pda.datastructures.Alignments:

[legend]

Collaboration diagram for broad.pda.datastructures.Alignments:

[legend]

|  |  |
| --- | --- |
| Public Member Functions | |
|  | Alignments (String ucsc) |
|  | |
|  | Alignments (String chr, int start, int end) |
|  | |
|  | Alignments (String chr, int start, int end, Strand strand) |
|  | |
|  | Alignments (String chr, int start, int end, String strand) |
|  | |
|  | Alignments (String chr, int start, int end, Strand strand, String name) |
|  | |
|  | Alignments (String chr, String start, String end) |
|  | |
|  | Alignments (Annotation a) |
|  | |
|  | Alignments (String fileName, boolean fromFileName) |
|  | |
|  | Alignments (LightweightGenomicAnnotation a) |
|  | |
|  | Alignments (Alignment record) |
|  | |
|  | Alignments (nextgen.core.alignment.Alignment record) |
|  | |
| double | getCountScore () |
|  | |
| List< Double > | getScores () |
|  | |
| String | getSequence (Sequence chr, boolean repeatMask) throws Exception |
|  | |
| String | getSequence (String genomeDir) throws Exception |
|  | |
| void | setCountScore (double score) |
|  | |
| void | setLine (String line) |
|  | |
| void | setScores (List< Double > values) |
|  | |
| void | addScore (double score) |
|  | |
| double | getScore () |
|  | |
| String | toString () |
|  | |
| String | toFileName () |
|  | |
| String | toUCSC () |
|  | |
| String | toStringNum () |
|  | |
| boolean | overlapsCollection (Collection< Alignments > c) |
|  | |
| boolean | overlapsAtAll (Alignments align) |
|  | |
| boolean | overlapsAtAll (Set< Alignments > set) |
|  | |
| boolean | within (Alignments bigger) |
|  | |
| int | length () |
|  | |
| boolean | containedWithin (Alignments align) |
|  | |
| boolean | containedWithin (Alignments interval, Alignments gene) |
|  | |
| Alignments | merge (Alignments a1, Alignments a2) |
|  | |
| Alignments | merge (Alignments a1) |
|  | |
| int | getMidPoint () |
|  | |
| String | toStringWithScores () |
|  | |
| Public Member Functions inherited from broad.core.annotation.BasicGenomicAnnotation | |
|  | BasicGenomicAnnotation (String name) |
|  | |
|  | BasicGenomicAnnotation (String name, String chr, int start, int end) |
|  | |
|  | BasicGenomicAnnotation (String name, String chr, int start, int end, Strand strand) |
|  | |
|  | BasicGenomicAnnotation (LightweightGenomicAnnotation ga) |
|  | |
|  | BasicGenomicAnnotation (Annotation ga) |
|  | |
|  | BasicGenomicAnnotation (GenomicAnnotation ga) |
|  | |
|  | BasicGenomicAnnotation (String[] info) |
|  | |
| int | getLength () |
|  | |
| String | toString () |
|  | |
| String | getId () |
|  | |
| void | setId (String id) |
|  | |
| void | reduceToDifference (GenomicAnnotation other) |
|  | |
| List< Annotation > | disect (Annotation a) |
|  | |
| List< Annotation > | disect (List<?extends Annotation > disectors) |
|  | |
| boolean | isFlankedBy (TwoSubjectAnnotation twoSubjectAnnotation, int buffer) |
|  | |
| int | lastIdxWhereListSearchBroke () |
|  | |
| Sequence | getSequence () |
|  | |
| void | setSequence (Sequence sequence) |
|  | |
| void | setOrientation (boolean orientation) |
|  | |
| void | setThreePrimeBuffer (int bufferSize) |
|  | |
| void | setFivePrimeBuffer (int bufferSize) |
|  | |
| int | getFivePrimeBases () |
|  | |
| int | getThreePrimeBases () |
|  | |
| int | compareTo (GenomicAnnotation arg0) |
|  | |
| int | getOrientedEnd () |
|  | |
| int | getOrientedStart () |
|  | |
| void | addBlock (String name, int start, int end) |
|  | |
| boolean | mayHaveBlocks () |
|  | |
| Public Member Functions inherited from broad.core.annotation.BasicLightweightAnnotation | |
|  | BasicLightweightAnnotation () |
|  | |
|  | BasicLightweightAnnotation (String chr, int start, int end) |
|  | |
|  | BasicLightweightAnnotation (String chr, int start, int end, String orientation) |
|  | |
|  | BasicLightweightAnnotation (String chr, int start, int end, Strand orientation, String name) |
|  | |
|  | BasicLightweightAnnotation (String chr, int start, int end, String orientation, double Scr) |
|  | |
|  | BasicLightweightAnnotation (String chr, String start, String end) |
|  | |
|  | BasicLightweightAnnotation (LightweightGenomicAnnotation annotation) |
|  | |
|  | BasicLightweightAnnotation (Annotation annotation) |
|  | |
| String | getChromosomeString () |
|  | |
| int | getDistanceTo (LightweightGenomicAnnotation other) |
|  | |
| double | getExtraScore (int i) |
|  | |
| List< Double > | getExtraScores () |
|  | |
| String | getLocationString () |
|  | |
| long | getMiddle () |
|  | |
| int | getOverlap (LightweightGenomicAnnotation other) |
|  | |
| double | getScore () |
|  | |
| void | setReversedOrientation (boolean isInReversedOrientation) |
|  | |
| void | setScore (double score) |
|  | |
| boolean | almostEqual (LightweightGenomicAnnotation other, int fudge) |
|  | |
| boolean | contains (LightweightGenomicAnnotation other) |
|  | |
| boolean | inReversedOrientation () |
|  | |
| boolean | overlaps (Collection<?extends Annotation > others, int buffer) |
|  | |
| boolean | overlaps (Collection<?extends Annotation > others) |
|  | |
| void | addExtraScore (double score) |
|  | |
| void | removeExtraScores () |
|  | |
| void | setBoundariesFromAnnoations (List<?extends GenomicAnnotation > annotations) |
|  | |
| void | stitchTo (LightweightGenomicAnnotation other) |
|  | |
| void | takeIntersection (LightweightGenomicAnnotation other) |
|  | |
| void | takeUnion (LightweightGenomicAnnotation other) |
|  | |
| LightweightGenomicAnnotation | intersect (LightweightGenomicAnnotation other) |
|  | |
| Collection<?extends Annotation > | intersect (Collection<?extends Annotation > annotations) |
|  | |
| int | hashCode () |
|  | |
| int | length () |
|  | |
| String | toUCSC () |
|  | |
| String | toString () |
|  | |
| int | compareTo (LightweightGenomicAnnotation b) |
|  | |
| void | setOrientation (String orientation) |
|  | |
| String | getChromosome () |
|  | |
| void | setChromosome (String chr) |
|  | |
| Public Member Functions inherited from broad.core.annotation.LightweightGenomicAnnotation | |
| int | getStart () |
|  | |
| int | getEnd () |
|  | |
| String | getName () |
|  | |
| void | setName (String name) |
|  | |
| void | setStart (int start) |
|  | |
| void | setEnd (int end) |
|  | |
| Public Member Functions inherited from umms.core.annotation.Annotation | |
| int | getSAMStart () |
|  | |
| int | getSAMEnd () |
|  | |
| String | getReferenceName () |
|  | |
| String | getChr () |
|  | |
| Strand | getStrand () |
|  | |
| boolean | hasOrientation () |
|  | |
| boolean | isNegativeStrand () |
|  | |
| int | numBlocks () |
|  | |
| List<?extends Annotation > | getBlocks (boolean oriented) |
|  | |
| int | getSize () |
|  | |
| int | size () |
|  | |
| int | getLengthOnReference () |
|  | |
| boolean | isUnoriented () |
|  | |
| int | getReferenceCoordinateAtPosition (int positionInAnnotation) |
|  | |
| int | getPositionAtReferenceCoordinate (int referenceCoordinate) |
|  | |
| int | getReferenceCoordinateAtPosition (int positionInAnnotation, boolean ignoreOrientation) |
|  | |
| int | getPositionAtReferenceCoordinate (int referenceCoordinate, boolean ignoreOrientation) |
|  | |
| void | setOrientation (char orientation) |
|  | |
| void | setOrientation (Strand orientation) |
|  | |
| void | setOrientedStart (int orientedStart) |
|  | |
| void | setOrientedEnd (int orientedEnd) |
|  | |
| void | setReferenceName (String refName) |
|  | |
| boolean | equals (Annotation other) |
|  | |
| void | expand (int deltaStart, int deltaEnd) |
|  | |
| Annotation | trim (int deltaStart, int deltaEnd) |
|  | |
| void | shift (int delta) |
|  | |
| void | moveToCoordinate (int coordinateInReference) |
|  | |
| Annotation | copy () |
|  | |
| Annotation | minus (Annotation other) |
|  | |
| Annotation | minus (Collection<?extends Annotation > others) |
|  | |
| int | getDistanceTo (Annotation other) |
|  | |
| String | toBED () |
|  | |
| String | toBED (int r, int g, int b) |
|  | |
| String | toShortBED () |
|  | |
| String | toBEDGraph () |
|  | |
| String | getFullInfoString () |
|  | |
| boolean | overlaps (Annotation other, int buffer) |
|  | |
| boolean | overlaps (Annotation other) |
|  | |
| boolean | overlaps (Annotation other, int buffer, boolean considerOrientation) |
|  | |
| boolean | overlaps (Annotation other, boolean considerOrientation) |
|  | |
| boolean | overlapsStranded (Annotation other) |
|  | |
| int | getOverlap (Annotation other) |
|  | |
| boolean | contains (Annotation other) |
|  | |
| Annotation | union (Annotation other) |
|  | |
| Annotation | intersect (Annotation other) |
|  | |
| List< Annotation > | intersect (List<?extends Annotation > annotations) |
|  | |
| int | compareToAnnotation (Annotation b) |
|  | |
| void | stitchTo (Annotation next) |
|  | |
| String | toCigar () |
|  | |
| boolean | fullyContains (Annotation annotation) |
|  | |
| Annotation | complement () |
|  | |
| boolean | equals (Annotation other, boolean useOrientation) |
|  | |
| Collection<?extends Annotation > | getSpliceConnections () |
|  | |
| int | getMidpoint () |
|  | |
| Public Member Functions inherited from broad.core.annotation.GenomicAnnotation | |
| void | setReversedOrientation (boolean isInReversedOrientation) |
|  | |
| void | setOrientation (String orientationString) |
|  | |
| Strand | getOrientation () |
|  | |
| String | getLocationString () |
|  | |
| List<?extends Annotation > | getBlocks () |
|  | |
| Public Member Functions inherited from broad.core.annotation.Feature | |
| String | getName () |
|  | |

|  |  |
| --- | --- |
| Static Public Member Functions | |
| static int | distanceBetween (Alignments align1, Alignments align2) |
|  | |
| static void | write (String save, Collection< Alignments > alignments) throws IOException |
|  | |
| static void | write (String save, Collection< Alignments > alignments, boolean sort) throws IOException |
|  | |
| static void | write (String save, Collection< Alignments > alignments, final int sortOnScore) throws IOException |
|  | |
| static void | write (BufferedWriter writer, Collection< Alignments > alignments) throws IOException |
|  | |
| static void | write (BufferedWriter writer, Collection< Alignments > alignments, boolean sort) throws IOException |
|  | |
| Static Public Member Functions inherited from broad.core.annotation.BasicLightweightAnnotation | |
| static Collection  < BasicLightweightAnnotation > | mergeAllOverlappers (TreeSet< BasicLightweightAnnotation > regions) |
|  | |
| static LightweightGenomicAnnotation | createFromUCSC (String ucsc) |
|  | |
| static List< Annotation > | stitchList (Collection<?extends Annotation > sortedList, int maxDistanceToStitch) |
|  | |

|  |  |
| --- | --- |
| Additional Inherited Members | |
| Protected Member Functions inherited from broad.core.annotation.BasicGenomicAnnotation | |
|  | BasicGenomicAnnotation () |
|  | |
| Protected Member Functions inherited from broad.core.annotation.BasicLightweightAnnotation | |
| void | setStart (String data) |
|  | |

## Constructor & Destructor Documentation

|  |  |  |  |  |  |
| --- | --- | --- | --- | --- | --- |
| broad.pda.datastructures.Alignments.Alignments | ( | String | *ucsc* | ) |  |

Here is the caller graph for this function:

|  |  |  |  |
| --- | --- | --- | --- |
| broad.pda.datastructures.Alignments.Alignments | ( | String | *chr*, |
|  |  | int | *start*, |
|  |  | int | *end* |
|  | ) |  |  |

|  |  |  |  |
| --- | --- | --- | --- |
| broad.pda.datastructures.Alignments.Alignments | ( | String | *chr*, |
|  |  | int | *start*, |
|  |  | int | *end*, |
|  |  | Strand | *strand* |
|  | ) |  |  |

|  |  |  |  |
| --- | --- | --- | --- |
| broad.pda.datastructures.Alignments.Alignments | ( | String | *chr*, |
|  |  | int | *start*, |
|  |  | int | *end*, |
|  |  | String | *strand* |
|  | ) |  |  |

|  |  |  |  |
| --- | --- | --- | --- |
| broad.pda.datastructures.Alignments.Alignments | ( | String | *chr*, |
|  |  | int | *start*, |
|  |  | int | *end*, |
|  |  | Strand | *strand*, |
|  |  | String | *name* |
|  | ) |  |  |

|  |  |  |  |
| --- | --- | --- | --- |
| broad.pda.datastructures.Alignments.Alignments | ( | String | *chr*, |
|  |  | String | *start*, |
|  |  | String | *end* |
|  | ) |  |  |

|  |  |  |  |  |  |
| --- | --- | --- | --- | --- | --- |
| broad.pda.datastructures.Alignments.Alignments | ( | Annotation | *a* | ) |  |

|  |  |  |  |
| --- | --- | --- | --- |
| broad.pda.datastructures.Alignments.Alignments | ( | String | *fileName*, |
|  |  | boolean | *fromFileName* |
|  | ) |  |  |

|  |  |  |  |  |  |
| --- | --- | --- | --- | --- | --- |
| broad.pda.datastructures.Alignments.Alignments | ( | LightweightGenomicAnnotation | *a* | ) |  |

Here is the call graph for this function:

|  |  |  |  |  |  |
| --- | --- | --- | --- | --- | --- |
| broad.pda.datastructures.Alignments.Alignments | ( | Alignment | *record* | ) |  |

|  |  |  |  |  |  |
| --- | --- | --- | --- | --- | --- |
| broad.pda.datastructures.Alignments.Alignments | ( | nextgen.core.alignment.Alignment | *record* | ) |  |

## Member Function Documentation

|  |  |  |  |  |  |
| --- | --- | --- | --- | --- | --- |
| void broad.pda.datastructures.Alignments.addScore | ( | double | *score* | ) |  |

|  |  |  |  |  |  |
| --- | --- | --- | --- | --- | --- |
| boolean broad.pda.datastructures.Alignments.containedWithin | ( | Alignments | *align* | ) |  |

Here is the call graph for this function:

|  |  |  |  |
| --- | --- | --- | --- |
| boolean broad.pda.datastructures.Alignments.containedWithin | ( | Alignments | *interval*, |
|  |  | Alignments | *gene* |
|  | ) |  |  |

Here is the call graph for this function:

|  |  |  |  |
| --- | --- | --- | --- |
| static int broad.pda.datastructures.Alignments.distanceBetween | ( | Alignments | *align1*, |
|  |  | Alignments | *align2* |
|  | ) |  |  |

static

Here is the call graph for this function:

|  |  |  |  |  |
| --- | --- | --- | --- | --- |
| double broad.pda.datastructures.Alignments.getCountScore | ( |  | ) |  |

|  |  |  |  |  |
| --- | --- | --- | --- | --- |
| int broad.pda.datastructures.Alignments.getMidPoint | ( |  | ) |  |

Here is the call graph for this function:

|  |  |  |  |  |
| --- | --- | --- | --- | --- |
| double broad.pda.datastructures.Alignments.getScore | ( |  | ) |  |

Implements broad.core.annotation.Feature.

Here is the caller graph for this function:

|  |  |  |  |  |
| --- | --- | --- | --- | --- |
| List<Double> broad.pda.datastructures.Alignments.getScores | ( |  | ) |  |

Here is the caller graph for this function:

|  |  |  |  |
| --- | --- | --- | --- |
| String broad.pda.datastructures.Alignments.getSequence | ( | Sequence | *chr*, |
|  |  | boolean | *repeatMask* |
|  | ) |  | throws Exception |

|  |  |  |  |  |  |
| --- | --- | --- | --- | --- | --- |
| String broad.pda.datastructures.Alignments.getSequence | ( | String | *genomeDir* | ) | throws Exception |

|  |  |  |  |  |
| --- | --- | --- | --- | --- |
| int broad.pda.datastructures.Alignments.length | ( |  | ) |  |

Returns
:   length of the Annotation; if it has blocks, the gaps between blocks are not included

Implements broad.core.annotation.LightweightGenomicAnnotation.

Here is the call graph for this function:

Here is the caller graph for this function:

|  |  |  |  |
| --- | --- | --- | --- |
| Alignments broad.pda.datastructures.Alignments.merge | ( | Alignments | *a1*, |
|  |  | Alignments | *a2* |
|  | ) |  |  |

Here is the call graph for this function:

|  |  |  |  |  |  |
| --- | --- | --- | --- | --- | --- |
| Alignments broad.pda.datastructures.Alignments.merge | ( | Alignments | *a1* | ) |  |

Here is the call graph for this function:

|  |  |  |  |  |  |
| --- | --- | --- | --- | --- | --- |
| boolean broad.pda.datastructures.Alignments.overlapsAtAll | ( | Alignments | *align* | ) |  |

Here is the call graph for this function:

Here is the caller graph for this function:

|  |  |  |  |  |  |
| --- | --- | --- | --- | --- | --- |
| boolean broad.pda.datastructures.Alignments.overlapsAtAll | ( | Set< Alignments > | *set* | ) |  |

Here is the call graph for this function:

|  |  |  |  |  |  |
| --- | --- | --- | --- | --- | --- |
| boolean broad.pda.datastructures.Alignments.overlapsCollection | ( | Collection< Alignments > | *c* | ) |  |

Here is the call graph for this function:

|  |  |  |  |  |  |
| --- | --- | --- | --- | --- | --- |
| void broad.pda.datastructures.Alignments.setCountScore | ( | double | *score* | ) |  |

|  |  |  |  |  |  |
| --- | --- | --- | --- | --- | --- |
| void broad.pda.datastructures.Alignments.setLine | ( | String | *line* | ) |  |

|  |  |  |  |  |  |
| --- | --- | --- | --- | --- | --- |
| void broad.pda.datastructures.Alignments.setScores | ( | List< Double > | *values* | ) |  |

|  |  |  |  |  |
| --- | --- | --- | --- | --- |
| String broad.pda.datastructures.Alignments.toFileName | ( |  | ) |  |

Here is the call graph for this function:

|  |  |  |  |  |
| --- | --- | --- | --- | --- |
| String broad.pda.datastructures.Alignments.toString | ( |  | ) |  |

Here is the call graph for this function:

Here is the caller graph for this function:

|  |  |  |  |  |
| --- | --- | --- | --- | --- |
| String broad.pda.datastructures.Alignments.toStringNum | ( |  | ) |  |

Here is the call graph for this function:

|  |  |  |  |  |
| --- | --- | --- | --- | --- |
| String broad.pda.datastructures.Alignments.toStringWithScores | ( |  | ) |  |

Here is the call graph for this function:

|  |  |  |  |  |
| --- | --- | --- | --- | --- |
| String broad.pda.datastructures.Alignments.toUCSC | ( |  | ) |  |

Similar to getLocationString()

See also
:   getLocationString and provided only for backwards compatibility.

Implements broad.core.annotation.LightweightGenomicAnnotation.

Here is the call graph for this function:

|  |  |  |  |  |  |
| --- | --- | --- | --- | --- | --- |
| boolean broad.pda.datastructures.Alignments.within | ( | Alignments | *bigger* | ) |  |

Here is the call graph for this function:

|  |  |  |  |
| --- | --- | --- | --- |
| static void broad.pda.datastructures.Alignments.write | ( | String | *save*, |
|  |  | Collection< Alignments > | *alignments* |
|  | ) |  | throws IOException |

static

Here is the caller graph for this function:

|  |  |  |  |
| --- | --- | --- | --- |
| static void broad.pda.datastructures.Alignments.write | ( | String | *save*, |
|  |  | Collection< Alignments > | *alignments*, |
|  |  | boolean | *sort* |
|  | ) |  | throws IOException |

static

Write alignments

Parameters
:   |  |  |
    | --- | --- |
    | save |  |
    | alignments |  |
    | sort | sort by genomic position |

Exceptions
:   |  |  |
    | --- | --- |
    | IOException |  |

Here is the call graph for this function:

|  |  |  |  |
| --- | --- | --- | --- |
| static void broad.pda.datastructures.Alignments.write | ( | String | *save*, |
|  |  | Collection< Alignments > | *alignments*, |
|  |  | final int | *sortOnScore* |
|  | ) |  | throws IOException |

static

Write alignments sorted by score i

Parameters
:   |  |  |
    | --- | --- |
    | args |  |

Here is the call graph for this function:

|  |  |  |  |
| --- | --- | --- | --- |
| static void broad.pda.datastructures.Alignments.write | ( | BufferedWriter | *writer*, |
|  |  | Collection< Alignments > | *alignments* |
|  | ) |  | throws IOException |

static

Here is the call graph for this function:

|  |  |  |  |
| --- | --- | --- | --- |
| static void broad.pda.datastructures.Alignments.write | ( | BufferedWriter | *writer*, |
|  |  | Collection< Alignments > | *alignments*, |
|  |  | boolean | *sort* |
|  | ) |  | throws IOException |

static

---

The documentation for this class was generated from the following file:

- java/broad/pda/datastructures/Alignments.java


---

Generated on Fri Aug 15 2014 15:10:58 for ESAT by  

 1.8.7
